# A pH-Gated Functionalized Hollow Mesoporous Silica Delivery System for Photodynamic Sterilization in *Staphylococcus aureus* Biofilm

**DOI:** 10.3390/ma15082815

**Published:** 2022-04-12

**Authors:** Nanxin Zhao, Rongfeng Cai, Yuting Zhang, Xiaoli Wang, Nandi Zhou

**Affiliations:** The Key Laboratory of Carbohydrate Chemistry and Biotechnology, Ministry of Education, School of Biotechnology, Jiangnan University, Wuxi 214122, China; 6190203015@jiangnan.edu.cn (N.Z.); 8202107006@jiangnan.edu.cn (R.C.); zhangyuting@jiangnan.edu.cn (Y.Z.); wangxiaoli@jiangnan.edu.cn (X.W.)

**Keywords:** bacteria biofilm, pH-gated delivery, hollow mesoporous silica, photodynamic sterilization, *Staphylococcus aureus*

## Abstract

Multidrug-resistant bacteria are increasing, particularly those embedded in microbial biofilm. These bacteria account for most microbial infections in humans. Traditional antibiotic treatment has low efficiency in sterilization of biofilm-associated pathogens, and thus the development of new approaches is highly desired. In this study, amino-modified hollow mesoporous silica nanoparticles (AHMSN) were synthesized and used as the carrier to load natural photosensitizer curcumin (Cur). Then glutaraldehyde (GA) and polyethyleneimine (PEI) were used to seal the porous structure of AHMSN by the Schiff base reaction, forming positively charged AHMSN@GA@PEI@Cur. The Cur delivery system can smoothly diffuse into the negatively charged biofilm of *Staphylococcus aureus* (*S. aureus*). Then Cur can be released to the biofilm after the pH-gated cleavage of the Schiff base bond in the slightly acidic environment of the biofilm. After the release of the photosensitizer, the biofilm was irradiated by the blue LED light at a wavelength of 450 nm and a power of 37.4 mV/cm^2^ for 5 min. Compared with the control group, the number of viable bacteria in the biofilm was reduced by 98.20%. Therefore, the constructed pH-gated photosensitizer delivery system can efficiently target biofilm-associated pathogens and be used for photodynamic sterilization, without the production of antibiotic resistance.

## 1. Introduction

Since the wide transfer of antibiotic-resistance genes in bacteria caused by the irrational use of antibiotics, the prevalence of clinical and environmental multidrug-resistant (MDR) bacteria has increased significantly, causing approximately 700,000 deaths globally each year [1,2]. Besides antibiotic-resistance genes, bacteria can form biofilms for the additional resistance of antibiotic exposure [2]. Biofilms are a complex microbial community composed of bacterial cells, water, and a mass of voluntary extracellular polymers (EPS) [3]. Biofilm-associated cells exhibit greater resistance to environmental challenges, and research shows that 65–80% of microbial infections are associated with biofilms [3,4,5]. Many bacteria including some common pathogens have the ability to form biofilms, such as *Escherichia coli*, *Klebsiella pneumoniae*, *Pseudomonas aeruginosa* and *Staphylococcus aureus* [6]. Moreover, biofilms can occur on medical devices, such as contact lenses, catheters, pacemakers, and even on the skin or mucosal surfaces of the respiratory tract and digestive tract, causing diseases in the related part of the body [7,8]. When exposed to adverse conditions, such as starvation and antibiotics, free bacteria initiate the formation of biofilms through the activation of the related genes. Bacterial cells within biofilms produce acidic by-products, thereby biofilms are slightly acidic environments [5,9,10,11]. After the formation of biofilms, bacteria can survive various physicochemical attacks, such as ultraviolet light, heavy metals, acidity, etc. Particularly, biofilm-associated bacteria can limit or completely block the penetration of antibiotics and resist the immune system of hosts, which makes the recurrence rate high and removing biofilm infections difficult [9,12,13]. Therefore, the development of novel anti-biofilm strategies is greatly desired.

Recently, various nanomaterials have been applied in antimicrobial areas, including direct sterilization or helping drugs penetrate biofilms and expose biofilm-associated bacteria to drugs, and therefore, improving the efficiency of drug treatment. For example, silver nanoparticles, zinc nanoparticles and some other metal and metal oxide nanoparticles have broad-spectrum antibacterial effects on bacterial cells [14,15]. Carbon-based nanomaterials can produce a photothermal effect to kill bacteria [16]. Some nanomaterials, such as mesoporous silica, liposomes, and other polymeric nanomaterials can be used as drug carriers [17]. In order to effectively penetrate biofilm and control infection, the ideal diameter range of nanoparticles is from 5 nm to 500 nm [18]. Research shows that positively charged nanoparticles are more likely to interact with negatively charged EPS to penetrate biofilms, and thus show stronger toxicity to negatively charged bacterial cells [19,20,21]. Mesoporous silicon nanoparticles (MSN) are one of the most promising nanocarriers for drug delivery due to their high loading capacity, high biocompatibility, adjustable pore size and particle size, easy synthesis, and easy functional modification [18,22,23,24]. In recent years, MSN has also been employed in biofilm removal [25,26,27,28,29]. The easy functional modification of MSN enables it to combine with other materials to exhibit various properties, which makes it a promising carrier in the treatment of biofilm infections. Moreover, gated materials can be constructed on MSN to control the release of substances in the mesopore under different conditions [22,30]. Among them, pH is a common stimulus. Different substances, such as amines, polymers and proteins have been modified on the surface of MSN to control the release of cargoes under different pH conditions [31,32].

To avoid the development of drug resistance, there is an urgent need to develop treatments that can effectively remove biofilm bacteria without making the bacteria resistant. Antimicrobial therapy, photodynamic therapy (PDT) and photothermal therapy (PTT) based on bioactive materials show increasing potential in this regard [16]. PDT is based on the fact that the irradiation of photosensitizer will stimulate the production of reactive oxygen species (ROS), which have a killing effect on bacterial cells [33]. At present, PDT has been proven to have great potential in the treatment of oral infections caused by biofilms [34,35]. Curcumin (Cur) is a natural active substance extracted from turmeric and turmeric rhizomes, with anti-inflammatory, antioxidant, anti-microbial activities, and photosensitivity [36,37]. Many studies have proven that curcumin can trigger the PDT effect to generate ROS under the irradiation of blue LED light, which can sterilize and remove biofilm [38,39]. Due to the poor water solubility of curcumin, the effective bioavailability of curcumin is insufficient, resulting in a poor curative effect. However, using nanomaterials as the carrier of curcumin can increase the intake and curative effect of curcumin [40].

In order to develop a treatment that can effectively inhibit bacteria in mature biofilms and avoid bacterial resistance, a positively charged AHMSN@GA@PEI@Cur nano-delivery system was designed and its performance was evaluated by using the biofilm-associated pathogen *S. aureus* as an example. The complex can easily penetrate the biofilm, and then pH-gated release of curcumin was achieved in the slightly acidic microenvironment of the biofilm. The released curcumin with both bactericidal and photosensitizing activities effectively killed the bacteria in the biofilm.

## 2. Materials and Methods

### 2.1. Materials and Regents

Tetraethylorthosilicate (TEOS), and polyethyleneimine (PEI) were obtained from Macklin Biochemical Technology Co., Ltd. (Shanghai, China). Ammonia water (NH_3_·H_2_O, 14.84 M), triethanolamine, 3-triethoxysilylpropylamine (APTE), anhydrous sodium carbonate (Na_2_CO_3_), curcumin (Cur), glutaraldehyde (GA), and phosphate buffered saline (PBS), were purchased from Sinopharm Chemical Reagent Co., Ltd. (Shanghai, China). Dialysis membrane and cetyltrimethylammonium bromide (CTAB) were obtained from Sangon Biotech Co., Ltd. (Shanghai, China). Tryptone, yeast extract was obtained from Oxoid Ltd. (Basingstoke, New Hampshire, UK). Agar was obtained from Beijing Jin Ming Biotechnology Co., Ltd. (Beijing, China) Trypticase Soy Broth was obtained from Qingdao Hope Bio-Technology Co., Ltd. (Qindao, China) *S. aureus* (ATCC29213) is a laboratory collection standard strain. All other chemicals in the experiment are analytical reagents and can be used without further purification.

### 2.2. Synthesis of Functionalized Hollow Mesoporous Silica Nanoparticles Loaded with Curcumin (AHMSN@GA@PEI@Cur)

Hollow mesoporous silica nanoparticles (HMSN) were synthesized by referring to the reported method with slight adjustment [41]. First, NH_3_·H_2_O (1.6 mL) was added to a mixture of absolute ethanol (71.4 mL) and water (1.6 mL) to create an alkaline environment. TEOS (2 mL) was then added to the above mixture and reacted at room temperature for 1 h. The reaction solution was centrifuged (10,062 g, 15 min) and washed twice with ethanol and ultrapure water to collect the produced SiO_2_, which was suspended in 40 mL water for subsequent use.

Next, a mixture of 1.5 g CTAB, 53.4 μL TEA and 64.5 mL water was vigorously stirred at 80 °C to dissolve completely. Then 30 mL of the above SiO_2_ suspension was added to the solution and stirred continuously for 20 min, then 450 μL of TEOS was added and incubated for 1 h. The mixture was cooled to 50 °C, followed by the addition of 9 mL saturated Na_2_CO_3_ solution to etch the silica sphere template for 2 h. The hollow silica spheres (HSS) were obtained by centrifugation (10,062 g, 15 min) and washed twice with ethanol and ultrapure water, respectively. Subsequently, the obtained HSS and 2 g NH_4_NO_3_ were dispersed in 100 mL ethanol, then stirred at 60 °C for 2 h to remove CTAB. HMSN was obtained after centrifugation (10,062 g, 15 min). This process was repeated twice to ensure the complete removal of the CTAB.

To prepare amino-functionalized hollow mesoporous silica nanoparticles (AHMSN), 30 mg of HMSN and 15 μL of APTE were dissolved in 30 mL ethanol, and the solution was stirred at 25 °C for 24 h. After the reaction, the samples were washed twice with water and ethanol to obtain AHMSN.

Next, to load curcumin in AHMSN, 20 mg AHMSN was added into 5 mL Cur ethanol solution (4 mg/mL). After ultrasonic dispersion, the mixture was incubated at room temperature for 24 h in the dark. Then the supernatant was removed after centrifugation, and the collected AHMSN loaded curcumin (AHMSN@Cur) was washed three times with ethanol.

Finally, 20 mg AHMSN@Cur, 12 mg of 25% GA and 20 mg of 25% PEI were added in 5 mL of deionized water (pH 8.0). The mixture was shaken at 40 °C for 4 h. After centrifugation, the sediment was washed with deionized water three times to obtain AHMSN@GA@PEI@Cur.

### 2.3. Characterization of the Nanomaterials

The properties and structures of AHMSN and AHMSN@GA@PEI@Cur were analyzed by a JEM-2100 transmission electron microscope (JEOL Ltd., Musashino, Japan.), ZEN3700 nanoparticle size and ZETA potential analyzer (Malvern Instruments Co., LTD, Malvern, UK), and NEXUS Fourier transform Characterization by an infrared spectrometer (Nicolic Instruments, Inc., Madison, WI, USA). The concentration of Cur was measured with an Enspire 2300 Multi-Label Detection System (Perkin Elmer Ltd., Norwalk, CT, USA).

### 2.4. pH-Triggered Release In Vitro

To study the pH-responsive release behavior of the nano-delivery system in vitro, 1 mg AHMSN@GA@PEI@Cur was dispersed into 1 mL PBS buffer with different pH (pH 5.0, 6.5 and 7.4), and placed in a half permeable membrane (molecular weight cut-off 3.5 kDa). Then the half permeable membrane was placed in a 50 mL centrifuge tube containing 19 mL PBS of the same pH condition and incubated at 37 °C under 100 rpm rotation. An aliquot of the solution in the centrifuge tube (1 mL) was withdrawn at a regular time interval. An equal volume of PBS buffer was refilled into the tube. The amount of Cur released in the withdrawn solution was measured by the fluorescence method. All assays were performed in triplicate. The mean and standard deviation were calculated by Origin 2021.

### 2.5. Preparation of S. aureus Biofilm

The activated *S. aureus* was inoculated into 50 mL of sterile TSB medium and cultured overnight with shaking at 25 °C and 220 rpm. After adding 500 μL *S. aureus* (~10^8^ CFU/mL) to a sterile 6-well plate, 5 mL TSB medium was added to each well, and a sterile coverslip was placed on each well. The bacteria were cultured at 37 °C for 2 days. The coverslips and plate were washed three times with sterilized PBS to remove free bacteria.

### 2.6. Photodynamic Treatment of Biofilm-Associated S. aureus and the Evaluation of the Sterilization Effect

Five milliliters of AHMSN@GA@PEI@Cur, AHMSN and free Cur in PBS (pH 7.4) were added to the wells of the cultured plate with biofilm, respectively, and incubated at 37 °C and 100 rpm rotation for 24 h in the dark. After the incubation, each well was irradiated with a blue LED light at 450 nm, 37.4 mW/cm^2^ for 5 min. The incubation solution was removed and then the coverslips were washed three times with sterile PBS. The coverslips were taken out and placed in a centrifuge tube with 5 mL sterile PBS, then the biofilm-associated bacteria attached to the coverslips were dispersed into PBS solution by ultrasound for 30 min. After dilution, the bacterial solution was spread on LB agar plates, the colonies were counted after overnight culture and the concentration of the bacteria was calculated. All assays were performed in triplicate. The mean and standard deviation were calculated by Origin 2021.

## 3. Results

### 3.1. The Design of the Nano-Delivery System That Can Kill Biofilm-Associated Bacteria

**Scheme 1:**
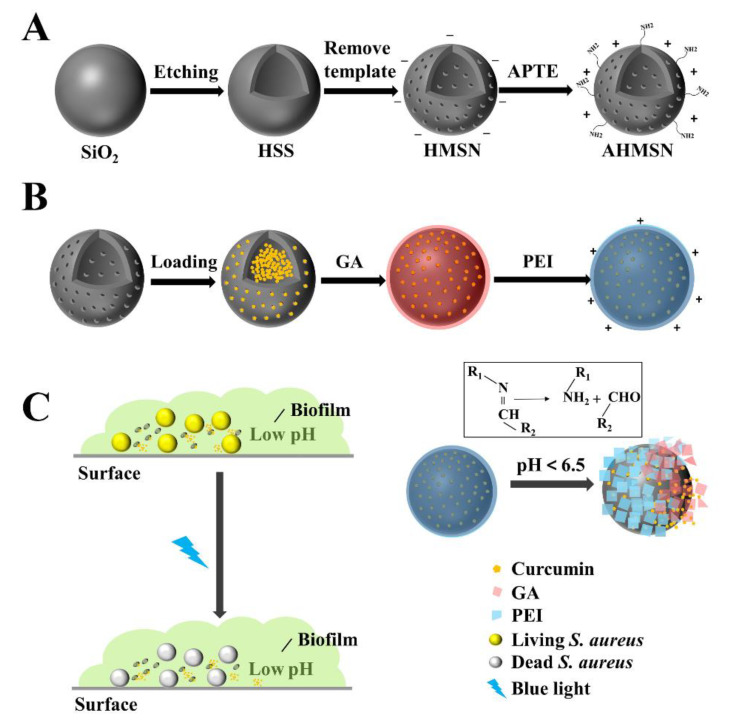
Schematic diagrams of (**A**) Synthetic route of AHMSN; (**B**) Preparation of AHMSN@GA@PEI@Cur; and (**C**) Application of AHMSN@GA@PEI@Cur in pH-gated biofilm-targeted sterilization.

The processes of the synthesis of the material and the application to *S. aureus* biofilm are shown in Scheme 1.

The solid silicon sphere is first synthesized by the sol-gel method, then HSS is obtained by etching. After the removal of the template CTAB, the HMSN is obtained. After further modification with ATPE, the amino group is attached to the surface of the HMSN to form the AHMSN, which is used as the carrier of the drug. The synthesis of the drug carrier AHMSN is shown in Scheme 1A.

The AHMSN is then loaded with Cur. To seal Cur in the porous structure of AHMSN, GA is used as a crosslinking agent to modify PEI on the surface of AHMSN through Schiff base bonds. The nano-delivery system AHMSN@GA@PEI@Cur is, therefore, prepared. The loading of the drug and the sealing of the delivery system are shown in Scheme 1B.

To treat the biofilm-associated *S. aureus*, AHMSN@GA@PEI@Cur is introduced and allowed to diffuse into the biofilm through electrostatic attraction between the positively charged AHMSN@GA@PEI@Cur and the negatively charged biofilm. Then the Schiff base bond cleaves in the low pH environment of the biofilm, and the photosensitive drug Cur diffuses into the biofilm microenvironment. Under the irradiation of the blue LED light at 450 nm, Cur exerts a photodynamic effect to synergistically kill the biofilm-associated bacteria. The application of the nano-delivery system is shown in Scheme 1C.

### 3.2. Characterization of the Nanomaterials

**Figure 1:**
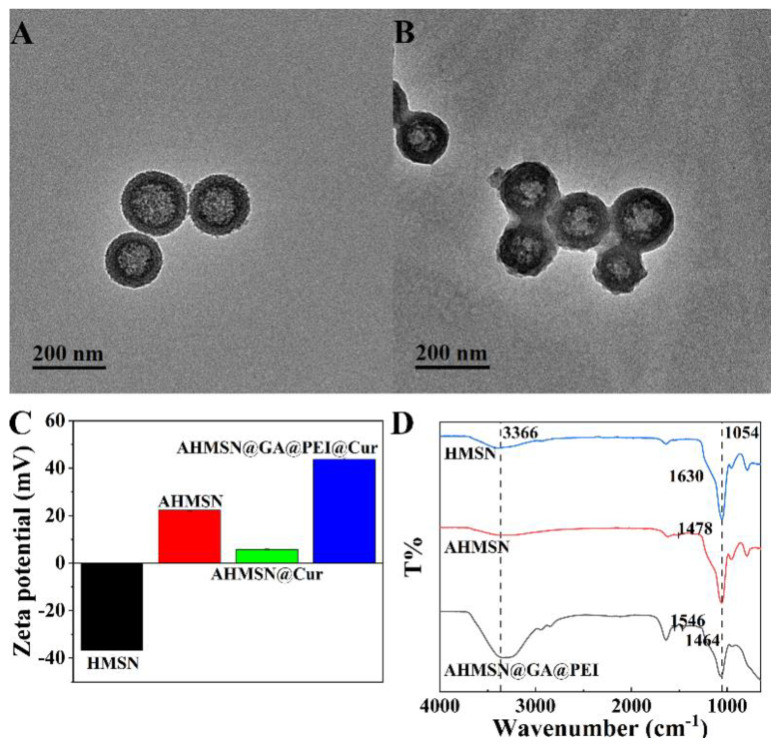
Characterizations of the synthesis and modification of the nanomaterials. (**A**) TEM image of AHMSN, the diameter of AHMSN is about 155 nm; (**B**) TEM image of AHMSN@GA@PEI, the diameter of AHMSN@GA@PEI is about 176 nm; (**C**) Zeta potentials of HMSN (-36.73 mV), AHMSM (22.27 mV), AHMSN@Cur (5.73 mV) and AHMSN@GA@PEI@Cur (43.80 mV); (**D**) FT-IR spectra of HMSN, AHMSN and AHMSN@GA@PEI.

The structure and property of the synthesized nanomaterials were characterized by TEM, DLS, Zeta potential analyzer, fluorescent spectroscopy and FT-IR. According to the TEM image shown in Figure 1A, the synthesized AHMSN has a regular hollow spherical shape with an average particle size of about 155 nm. The particle size distribution of AHMSN is shown in Figure S1 in the Supplementary Material. Porous structure on the surface of AHMSN can be clearly observed. Meanwhile, according to the TEM image shown in Figure 1B, after modification with GA and PEI, the average particle size of AHMSN@GA@PEI slightly increases to about 176 nm. The hydrated particle size of MSN is 186.03 nm, and after being modified with APTE and PEI, the hydrated particle size becomes 200.70 nm and 366.63 nm, respectively. The Polydispersity index (PDI) of MSN, AHMSN and AHMSN@GA@PEI are all less than 0.3. The detailed data of DLS are shown in Table 1.

Zeta potentials of the nanomaterials during the modification were measured. The potential of HMSN is −36.73 mV. After the modification of amino groups by APTE, the potential of AHMSN increases to 22.27 mV. After loading of Cur, the potential decreases to 5.73 mV. After modification of PEI by GA crosslinking, the potential of AHMSN@GA@PEI@Cur increases to 43.80 mV. The results are shown in Figure 1C.

In order to further verify the successful modification of HMSN, FT-IR characterization was carried out. HMSN, AHMSN, AHMSN@GA@PEI all have strong and broad Si–O–Si characteristic peaks at about 1054 cm^−1^. After modification with APTE, the obtained AHMSN has a typical -NH_2_ peak at 1478 cm^−1^. After modification with GA and PEI, There is a new peak at 1546 cm^−1^. In addition, after the modification of GA and PEI, the infrared spectrum shows obvious characteristic peaks at 3366 cm^−1^, 2945 cm^−1^ and 2848 cm^−1^, respectively. The FT-IR patterns are shown in Figure 1D.

The fluorescent method was used to monitor the loading of Cur, due to the fluorescent characteristics of Cur. The fluorescence emission spectrum of Cur was recorded with a microplate reader at the excitation wavelength of 425 nm (Figure S2). When the excitation wavelength is 425 nm, Cur has a maximum emission wavelength of 570 nm. Then the fluorescence intensities of different concentrations of Cur anhydrous ethanol solutions (5, 2, 1, 0.2, 0.1 μg/mL) were measured at the emission wavelength of 570 nm and used to draw the calibration curve (Figure S3). Based on this, the concentrations of Cur in the solution before and after loading were calculated by measuring the corresponding fluorescence intensities of the solution. The encapsulation efficiency (EE) can be assessed by the equation EE = (CT − CF)/CT × 100%, where CT is the total concentration of Cur fed, and CF is the concentration of non-encapsulated free Cur. The calculated EE% is 4.26%.

### 3.3. pH-Responsive Release of Curcumin In Vitro

**Figure 2:**
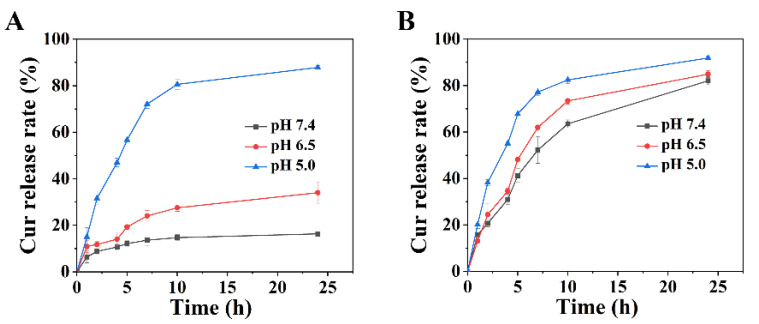
The release rate of Cur of (**A**) AHMSN@GA@PEI@Cur (The Cur release rates of AHMSN@GA@PEI@Cur at pH 7.4, pH 6.5, pH 5.0 are 16.39%, 34.04%, 87.88% at 24 h) and (**B**) AHMSN@Cur under different pH conditions.

The pH-responsive characteristic of the nano-delivery system was firstly investigated in the solution. Since the pH-responsive characteristic of the nano-delivery system is endowed by Schiff base bonded PEI encapsulation, the release rates of Cur of AHMSN@GA@PEI@Cur and unsealed AHMSN@Cur were compared. Figure 2 shows the in vitro release of Cur in PBS buffer with different pH (7.4, 6.5, 5.0). For AHMSN@GA@PEI@Cur, the release rate of Cur is apparently higher in an acidic environment. The release rate at pH 5.0 is 2.58-fold and 5.36-fold higher than those of pH 6.5 and pH 7.4, respectively. The details can be seen in Figure 2A.

As a comparison, the release rate of the unencapsulated AHMSN@Cur was also investigated under the same conditions. It is found that although the release rates slightly deviate at different pH, they are all at high levels without pH preference. After 24 h incubation in the solution at pH 5.0, the release rate of AHMSN@Cur reaches 91.86%. Meanwhile, the release rates reach 84.91% and 82.07% at pH 6.5 and pH 7.4, respectively, which are just slightly lower than that of pH 5.0. The data were shown in Figure 2B.

### 3.4. Treatment of Biofilm-Associated S. aureus

**Figure 3:**
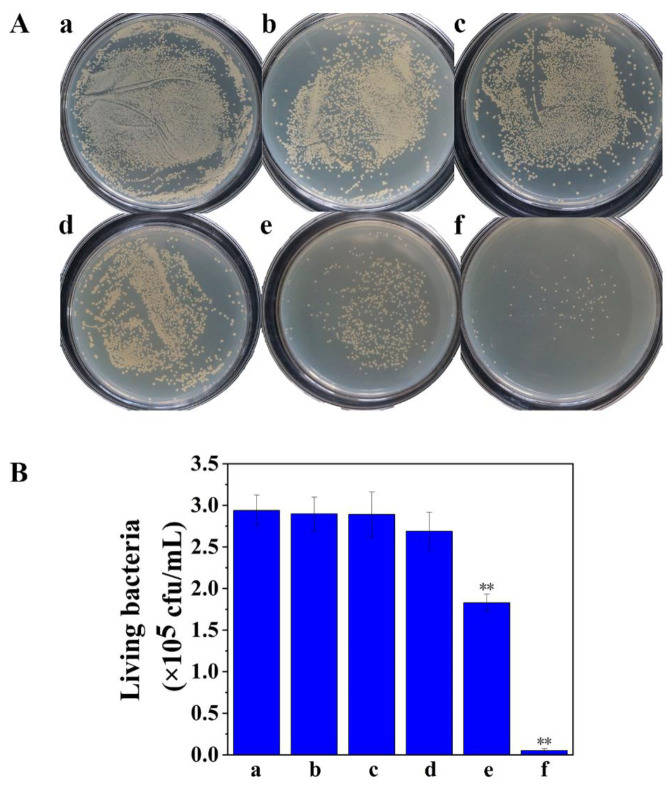
(**A**) The images of the cultured plates and (**B**) The concentration of viable bacteria of the samples treated under different conditions: (**a**) Control; (**b**) L; (**c**) AHMSN+L; (**d**) Cur+L; (**e**) AHMSN@Cur+L; (**f**) AHMSN@GA@PEI@Cur+L, (L: Blue light exposure for 5 min). The average viable bacteria concentrations are, respectively, 2.94 × 10^5^ cfu/mL, 2.90 × 10^5^ cfu/mL, 2.89 × 10^5^ cfu/mL, 2.69 × 10^5^ cfu/mL, 1.83 × 10^5^ cfu/mL, 5.3 × 10^3^ cfu/mL (** *p* < 0.05).

The pH-gated photodynamic sterilization of AHMSN@GA@PEI@Cur in *S. aureus* biofilm was performed. Once the biofilm is successfully formed, AHMSN@GA@PEI@Cur, AHMSN@Cur, AHMSN and Cur representing the experiment and control groups were put into 6-well plates and incubated under mild shaking for 24 h. After incubation, the cover glass was taken out and carefully washed, and the biofilm-associated bacteria adhered to the cover glass were dispersed into 5 mL sterile PBS by ultrasound. After being diluted to the appropriate concentration, the bacteria solution was spread on LB medium and cultured in a 37 °C incubator for 24 h. The living bacteria was calculated by plate counting method, to evaluate the effect of treatment in different groups on biofilm bacteria. The result of plate counting shows the sterilization effect of different treatment groups. Compared with the control group, the numbers of viable bacteria in the blue LED light irradiation (L) treated group and the AHMSN and irradiation (AHMSN+L) treated group decreased by 1.36% and 1.70%, respectively. Interestingly, the number of viable bacteria in the curcumin and irradiation (Cur+L) treated group showed only an 8.50% decrease. Compared with the Cur+L group, the viable bacteria number in the AHMSN@Cur and irradiation (AHMSN@Cur+L) treated group decreased by 37.76%. In the AHMSN@GA@PEI@Cur and irradiation (AHMSN@GA@PEI@Cur+L) treated group, the viable bacteria number decreased by 98.20%. Using IBM SPSS Statistics 26 software to conduct ANOVA analysis on the viable rate of *S. aureus* biofilm treated by experimental groups and control groups. The results showed that there were significant differences between AHMSN@GA@PEI@Cur and AHMSN@Cur treatment groups and the control group (*p* < 0.05). These details are shown in Figure 3.

**Figure 4:**
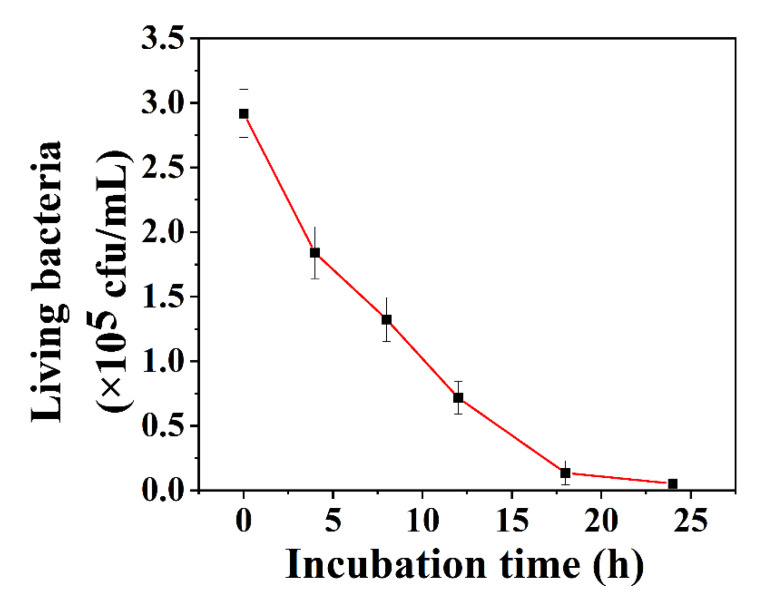
The change curve of living bacteria concentration after biofilm incubation with AHMSN@GA@PEI@Cur for different time (0 h, 4 h, 8 h, 12 h, 18 h, 24 h).

Furthermore, the effect of the incubation time of AHMSN@GA@PEI@Cur with the biofilms on the bactericidal rate was explored. As shown in Figure 4, with the increase in the incubation time, the number of viable bacteria declines quickly in the first 16 h and then slows down during further incubation.

## 4. Discussion

The characterization results of nanomaterials show the characteristics of the nano-delivery system and the change in its properties during the synthesis process. The TEM image of AHMSN indicates that it can be used as a carrier to load drugs into a delivery system. After being modified by APTE, GA and PEI, the diameter of AHMSN have a slight increase under TEM. During the modification with APTE, GA, and PEI, the hydrated particle size of the nanoparticles continuously increases, which illustrates the success of the modification. The polydispersity index (PDI) shows that the nanoparticles still have good dispersibility after modification. The zeta potentials of the nanomaterials have great changes in the process of functionalization. The zeta potential of HMSN is −36.73 mV, this is because of the hydroxyl groups on the surface of HMSN. Due to the amino groups of APTE, the zeta potential of AHMSN becomes 22.27 mV. The zeta potential of AHMSN@Cur decreases to 5.43 mV due to the hydroxyl group in the structure of Cur and then the zeta potential increases to 43.80 mV due to the free amino groups in PEI. The change of Zeta potential value during the modification process can prove that APTE and PEI have been successfully modified onto HMSN. The results of FT-IR show the characteristic peaks of nanomaterials. After modification with APTE, the obtained AHMSN has a typical -NH_2_ peak at 1478 cm^−1^. After modification with GA and PEI, the primary amino groups of AHMSN and PEI form Schiff base bonds with the aldehyde group in GA, and a C=N characteristic peak appears at 1546 cm^−1^. In addition, due to the abundant N-H bond in PEI and C-H bond and stretching aldehyde bond in GA, the infrared spectrum shows obvious characteristic peaks at 3366 cm^−1^, 2945 cm^−1^ and 2848 cm^−1^, respectively. The presence of these characteristic peaks is in accordance with those reported in the references [42,43,44]. In conclusion, the synthesis, modification and loading of the nano-delivery system were confirmed by TEM, DLS, Zeta potential analyzer, fluorescent spectroscopy and FT-IR methods.

The results of the pH-responsive release of curcumin in vitro show that the nano-diversity system has a great pH-controlled releasing performance. The difference in the release rate of AHMSN@GA@PEI@Cur under different pH conditions can mainly be attributed to the pH-sensitive C=N bond (Schiff base bond). In the previous studies, Schiff base bonds showed good pH response behavior [45,46]. The Schiff base bonded PEI encapsulation layer can effectively seal Cur in the nano-delivery system in neutral and basic pH environments while showing pH-responsive release in an acidic environment. As the bacterial biofilm microenvironment is slightly acidic, the pH-responsive nano-delivery system can meet the requirement.

The pH controlled nano-delivery system has been applied to the biofilm of *S. aureus*. The viability rate of the nano-delivery system showed that it had a good bactericidal performance. Compared with the control group, the numbers of viable bacteria in the blue LED light irradiation (L) treated group and the AHMSN and irradiation (AHMSN+L) treated group has no statistically significant difference. It indicates that blue light irradiation and AHMSN have a negligible killing effect on the biofilm-associated S. aureus and the poor bactericidal effect of free Cur can be attributed to its restricted permeation in biofilms. Because the carrier AHMSN can help Cur penetrate the biofilm and photodynamic sterilization effect, the viable bacteria number of AHMSN@Cur+L decreases by 37.76%. However, without a pH-gated encapsulation layer, Cur loaded in AHMSN can easily leak into the solution before the carrier penetrates into the biofilm, and the low Zeta potential of AHMSN@Cur also reduces the permeation rate of the carrier into the biofilm, so the viable bacteria number of AHMSN@Cur+L is higher than AHMSN@GA@PEI@Cur+L. Due to the positive charge of the AHMSN@GA@PEI@Cur nano-delivery system, it is easy to diffuse into the negatively charged biofilm. Meanwhile, the acidic microenvironment in the biofilm accelerates the cleavage of the Schiff base bond and the release of Cur. A remarkable photodynamic sterilization effect can be achieved after blue LED light irradiation. With the increase in incubation time, the nano-delivery system has a better bactericidal effect on biofilms.

PDT could be an avenue to explore to eradicate bacteria due to the surge of drug resistance. Many studies have used it to kill bacteria. Liu et al. conjugated lipopolysaccharide-bound antimicrobial peptides with protoporphyrin, a photosensitive agent, for imaging and photodynamic sterilization of Gram-negative strains [47]. PDT also had a good inhibitory effect on biofilm [48,49,50]. Compared with other reported substances, such as metal and metal oxide nanoparticles, antimicrobial peptides and natural extracts, which can also remove biofilm bacteria with a sterilization rate of 80% or more [14,51,52,53], the proposed material and method in this work exhibits the synergistic effect of sterilization and PDT of Cur. This mechanism makes the sterilization rate reach a higher level. The function of pH-controlled release enables it to trigger releasing Cur at a low pH, which fits the microenvironment of the biofilm. In the future, it will be interesting to focus research on the effects of nano-delivery systems on other biofilm-producing bacteria and applying the nano-delivery system to food processing.

## 5. Conclusions

In summary, well-dispersed AHMSN@GA@PEI nanomaterials were designed and synthesized. The nanomaterials can achieve pH-controlled release due to the Schiff base bond formed during the modification of GA and PEI. The nanomaterial was used as a nano-delivery system to load the natural photosensitive active substance curcumin. The nano-delivery system can penetrate the *S. aureus* biofilm and release curcumin into the acidic microenvironment of the biofilm. The sterilization rate of AHMSN@GA@PEI@Cur reached 98.20% under irradiation. The results show that the nano-delivery system has outstanding characteristics to treat biofilm-associated pathogens. It maintains stability in a normal neutral solution and specifically targets the acidic microenvironment of the biofilm. The sterilization efficiency is satisfactory, and thus it has great potential to be used against biofilm-associated infectious pathogens.

## Figures and Tables

**Table 1:** Hydrated particle size and PDI of MSN, AHMSN and AHMSN@GA@PEI.

Nanomaterials	Z-Ave (nm)	PDI
MSN	186.03	0.038
AHMSN	200.70	0.150
AHMSN@GA@PEI	366.63	0.264

## Data Availability

Not applicable.

## Supplementary Materials

The following supporting information can be downloaded at: https://www.mdpi.com/article/10.3390/ma15082815/s1, Figure S1: Particle size distribution of AHMSN; Figure S2: Fluorescence scanning spectrum of Cur; Figure S3: Standard curve of Cur in anhydrous ethanol.
