# Supplementary material for: A pH-Gated Functionalized Hollow Mesoporous Silica Delivery System for Photodynamic Sterilization in Staphylococcus aureus Biofilm
Source: Materials (Basel). 2022 Apr 12;15(8):2815. doi: 10.3390/ma15082815 (PMC9031160; doi:10.3390/ma15082815)
Supplement: Supplementary file 1 [file materials-15-02815-s001.zip › materials-1661508-supplementary.pdf]

materials

MDPI

Article

A pH-Gated Functionalized Hollow Mesoporous Silica Delivery System for Photodynamic Sterilization in *Staphylococcus aureus* Biofilm

Nanxin Zhao, Rongfeng Cai, Yuting Zhang, Xiaoli Wang and Nandi Zhou *

The Key Laboratory of Carbohydrate Chemistry and Biotechnology, Ministry of Education, School of Biotechnology, Jiangnan University, Wuxi 214122, China; 6190203015@jiangnan.edu.cn (N.Z.); 8202107006@jiangnan.edu.cn (R.C.); zhangyuting@jiangnan.edu.cn (Y.Z.);wangxiaoli@jiangnan.edu.cn (X.W.)
* Correspondence: zhounandi@jiangnan.edu.cn; Tel.: +86-510-85197831

Citation: Zhao, N.; Cai, R.; Zhang, Y.; Wang, X.; Zhou, N. A pH-Gated Functionalized Hollow Mesoporous Silica Delivery System for Photodynamic Sterilization in *Staphylococcus aureus* Biofilm. *Materials* **2022**, *15*, 2815. https://doi.org/10.3390/ma15082815

Academic Editor: Sandra Maria Fernandes Carvalho

Received: 16 March 2022
Accepted: 6 April 2022
Published: 12 April 2022

Publisher's Note: MDPI stays neutral with regard to jurisdictional claims in published maps and institutional affiliations.

Copyright: © 2022 by the authors. Submitted for possible open access publication under the terms and conditions of the Creative Commons Attribution (CC BY) license (https://creativecommons.org/licenses/by/4.0/).

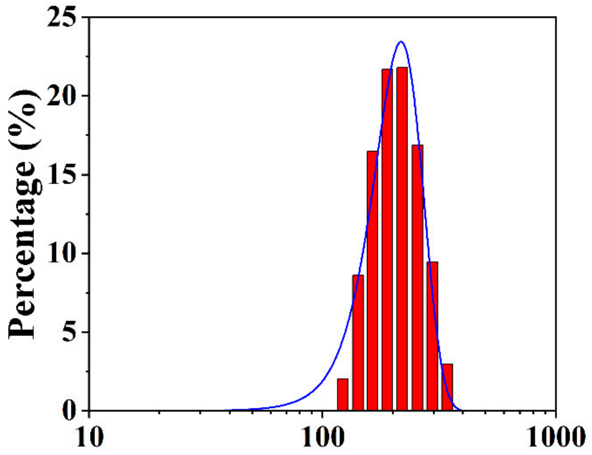

Figure S1. Particle size distribution of AHMSN.

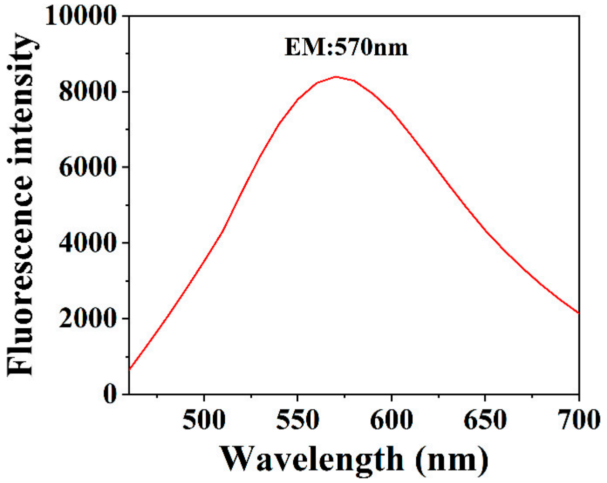

Figure S2. Fluorescence scanning spectrum of Cur.

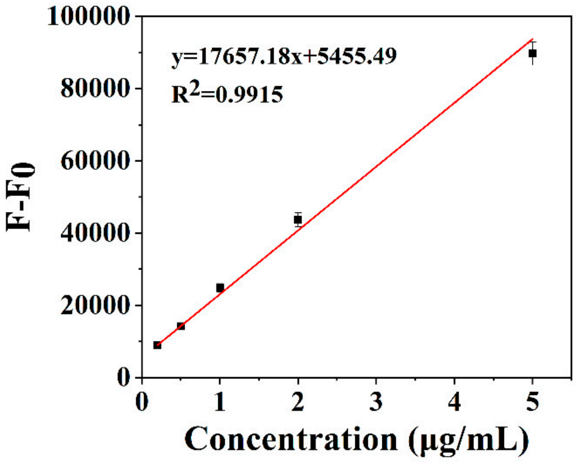

Figure S3. Standard curve of Cur in anhydrous ethanol.
